# neuTube 1.0: A New Design for Efficient Neuron Reconstruction Software Based on the SWC Format 1,2,3

**DOI:** 10.1523/ENEURO.0049-14.2014

**Published:** 2015-01-02

**Authors:** Linqing Feng, Ting Zhao, Jinhyun Kim

**Affiliations:** 1 Center for Functional Connectomics, Korea Institute of Science and Technology (KIST), Seoul, 136-791, Korea; 2 Janelia Research Campus, Howard Hughes Medical Institute, Ashburn, Virginia, 20147; 3 Neuroscience Program, University of Science and Technology, Daejeon, 305-350, Korea

**Keywords:** bioimage informatics, neuron tracing, SWC, user-friendly software

## Abstract

Brain circuit mapping requires digital reconstruction of neuronal morphologies in complicated networks. Despite recent advances in automatic algorithms, reconstruction of neuronal structures is still a bottleneck in circuit mapping due to a lack of appropriate software for both efficient reconstruction and user-friendly editing. Here we present a new software design based on the SWC format, a standardized neuromorphometric format that has been widely used for analyzing neuronal morphologies or sharing neuron reconstructions via online archives such as NeuroMorpho.org. We have also implemented the design in our open-source software called neuTube 1.0. As specified by the design, the software is equipped with parallel 2D and 3D visualization and intuitive neuron tracing/editing functions, allowing the user to efficiently reconstruct neurons from fluorescence image data and edit standard neuron structure files produced by any other reconstruction software. We show the advantages of neuTube 1.0 by comparing it to two other software tools, namely Neuromantic and Neurostudio. The software is available for free at http://www.neutracing.com, which also hosts complete software documentation and video tutorials.

## Abstract (precis)

Compared to other existing tools, the novel software we present has some unique features such as comprehensive editing functions and the combination of seed-based tracing and path searching algorithms, as well as their availability in parallel 2D and 3D visualization. These features allow the user to reconstruct neuronal morphology efficiently in a comfortable “What You See Is What You Get” (WYSIWYG) way.

## Significance Statement

Compared to other existing tools, the novel software we present has some unique features such as comprehensive editing functions and the combination of seed-based tracing and path searching algorithms, as well as their availability in parallel 2D and 3D visualization. These features allow the user to reconstruct neuronal morphology efficiently in a comfortable “What You See Is What You Get” (WYSIWYG) way.

## Introduction

Digital reconstruction, or tracing, of neuron morphologies from light microscope images is an important step in the mapping of brain circuits. In this task, the input is images and the output is usually a tree structure, which can be described by the SWC file format (Cannon et al., 1998). Although numerous neuron reconstruction software tools have been developed for producing SWC files (Meijering, 2010), none of them have taken full advantage of the SWC format to optimize the user interface for efficient and accurate reconstruction. An optimal user interface means that the user can interact with the software with minimal cognitive load, which requires data visualization to be clear and operations to be straightforward. In other words, with the visual information provided by the software, the user should be able to quickly figure out the underlying SWC model, how the model can be manipulated, and the results of manipulations. With these criteria in mind, we may identify disadvantages of many tracing software applications. For example, FARSIGHT (Luisi et al., 2011), which focuses on semi-automated reconstruction of neurons, does not provide intuitive low-level editing options to correct subtle errors. Simple Neurite Tracer, a popular plugin of Fiji (Longair et al., 2011), also lacks editing functions. Neurolucida, a mainstream commercial software tool, allows complete manual reconstruction of a neuron structure. However, it does not support neuron reconstruction in a 3D visualization window, despite the fact that 3D interaction has been demonstrated to improve both the speed and accuracy of the reconstruction procedure (Long et al., 2012). Some other popular software tools, such as Neuromantic (Myatt et al., 2012) and Neurostudio (Wearne et al., 2005), similarly lack advanced 3D editing functions. Vaa3D (Peng et al., 2010) provides innovative interactive neuron tracing functions in 3D, but these functions are not available in 2D to resolve dense or faint structures.

Here, we propose a new and comprehensive software design based on the SWC format as a solution to the diverse limitations of current tracing software. Based on this design, or, as we call it, the SWC framework, we have converted our previously reported software, neuTube (Kim et al., 2012), into a novel tool that enables efficient reconstruction by combining robust automatic tracing algorithms and versatile user-friendly editing functions in both 2D and 3D. This paper formally presents the redesigned software, neuTube 1.0, not only as a major upgrade of the previous release, but also as the first software to implement the SWC framework.

## Materials and Methods

### The SWC framework

**Figure 1:**
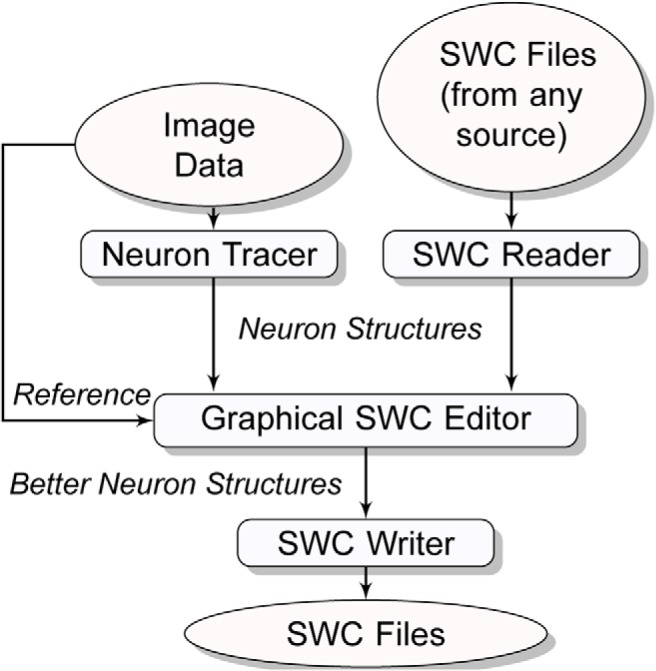
Workflow of reconstructing or editing a neuron structure in the SWC framework, which defines GUI software that takes either a raw image or an SWC file as input and generates an acceptable neuron structure through user interactions. The user can save the neuron structure into standard SWC files during or after reconstruction.

The overall layout of the SWC framework is shown in Figure 1. Software with this architecture takes a raw image or an SWC file as input and outputs a neuron structure satisfying the user. The design of the SWC framework follows the principle of “What You See Is What You Get” (WYSIWYG) (Peng et al., 2011), i.e., what the user is editing is explicitly visualized and no third-party viewer is needed to check the results. Therefore, the SWC framework consists of the following features: clear visualization of SWC structures, clear visualization of source images as reference data, explicit definition of operation units, and intuitive map from user inputs to editing operations. Except image visualization, these features are designed based on the SWC format, which describes a simple directed tree model, called the SWC model. Here, we described in detail how to construct operations on the SWC model by first defining the model in an abstract way.

#### Abstract definition of the SWC model

From a mathematical point of view, the SWC model can be defined as a set of nodes $\left\{n_{i}=(x_{i},y_{i},z_{i},r_{i},n_{j})\right|i=1,\ldots ,N,j=0,\ldots ,N,i\neq j,x_{i},y_{i},z_{i},r_{i}\in R\}$, where each node $n_{i}$ is a sphere with the center $\left(x_{i},y_{i},z_{i}\right)$ and the radius $r_{i}$. $n_{0}$ is an empty node for defining the root of a neuron structure, and $n_{j}$ is called the parent of $n_{i}$. An upstream path from $n_{i}$ to $n_{j}$ is an array of node $\left(n_{k_{1}},\ldots n_{k_{n}}\right)$, where $n_{k_{i+1}}$ is the parent of $n_{k_{i}}$, $k_{1}=i$, $k_{n}=j$. To form a valid tree structure of a neuron, no loop is allowed, i.e., there is at most one upstream path from one node to another. In this model, the basic structural unit is a node, which defines how we should design visualization and interactions.

#### SWC operation

Assuming $S_{1}$ and $S_{2}$ are two sets of nodes, the operation of a neuron structure is defined as

$$f(S_{1})=S_{2}$$

For example, $f\left(\left\{n_{1},\ldots ,n_{n}\right\}\right)=\phi$, where ϕ denotes the empty set, defines a removal operation. However, some operations may result in a new node set that forms an invalid neuron structure. How to construct a valid operation depends on the data structure describing the model. In our framework, we used a redundant tuple to store a node, which is $n=\left(G\left(n\right),P\left(n\right),C\left(n\right),S\left(n\right)\right)$, where $G\left(n\right)=\left(x\left(n\right),y\left(n\right),z\left(n\right),r(n)\right)$ defines that the node is located at $\left(x\left(n\right),y\left(n\right),z\left(n\right)\right)$ with radius r(n), $P\left(n\right)$ is the parent node of n, $C\left(n\right)$ is the first child of n and $S\left(n\right)$ is the next sibling of n. A sibling of n shares the same parent with n, i.e. $P\left(n\right)=P(S\left(n\right))$. The redundancy is designed to improve computational efficiency of visiting a node. For example, to query a child of a node, the program needs only to check its first child and traverse other children through the sibling link, while in a non-redundant representation where each node is only linked to its parent, the program may need to check every node in the tree.

Editing a node n is defined as changing the value of the corresponding tuple. We call any change on $G\left(n\right)$ a geometrical operation and any change on $P\left(n\right),C\left(n\right)$or $S\left(n\right)$ a structural operation. While a geometrical operation is straightforward, a structural operation may cause invalid neuron structures. For example, changing $P\left(n\right)$ alone may break the rule that $P\left(C(n\right))=n\;$ and $P\left(n\right)=P(S\left(n\right))$. To avoid this problem, we construct SWC operations at three levels in terms of operation complexity. The first level consists of three elementary operations linking a node n to another node n', as defined as follows

$$f_{p}(\{n\}|n'){=f}_{p}\left(\left\{\left(G(n),P\left(n\right),C\left(n\right),S\left(n\right)\right)\right\}|n'\right)=\;\left\{\left(G(n),n',C\left(n\right),S\left(n\right)\right)\right\}$$

$${f_{c}\left(\left\{n\right\}|n'\right)=f}_{c}\left(\left\{\left(G(n),P\left(n\right),C\left(n\right),S\left(n\right)\right)\right\}|n'\right)=\;\{\left(G(n),P(n),n',S\left(n\right)\right)\}$$

$$f_{s}\left(\left\{n\right\}|n^{'}\right)=f_{s}\left(\left\{\left(G(n),P\left(n\right),C\left(n\right),S\left(n\right)\right)\right\}|n'\right)=\;\{\left(G(n),P(n),C(n),n'\right)\}$$

At this level, structure validity is not guaranteed.

The second level consists of simple valid operations. Assuming $F_{p_{0}}\left(n\right)$ is the operation of setting the parent of n to $n_{0}$ (the empty node), if $C\left(P\left(n\right)\right)=n$, i.e. n is the first child of its parent, then

Fp0n=fs({n}|n0)∘fp{n}|n0∘fc({Pn}|Sn),  CPn=nfs({n}|n0)∘fp{n}|n0∘fs({S-1n}|Sn),  Otherwise

where f∘g denotes a composite operation and $S\left(S^{-1}\left(n\right)\right)=n$. To define an operation on a single node more explicitly, $F_{p_{0}}\left(n\right)$ is defined as a function of a node instead of a node set without adding any ambiguity.

The operation of setting a parent is

$$F_{p}\left(n|n'\right)=\;f_{c}(\{C(n^{'})\}|n)\circ f_{s}(\{n\}|C(n^{'}))\circ f_{p}(\{n\}|n')\circ F_{p_{0}}(n)$$

This operation also sets n as the first child of n'. In principle, this operation is sufficient for building all other operations. But in practice, it is useful to define one more operation, for setting a sibling:

$$F_{s}\left(n|n'\right)=\;f_{s}(\{n\}|n')\circ f_{s}(\{n'\}|S(n))\circ f_{p}(\{n'\}|P(n))\circ F_{p_{0}}(n')$$

The third level is a set of composition operations, which include any operation composed of the operations from the second level. At this level, we categorize the operations into two types, morphology-dependent and morphology-independent. An operation is morphology-dependent if the result of the operation depends on the positions or sizes of the nodes; otherwise it is morphology-independent.

Decomposing an operation into elementary operations helps guarantee the validity of neuron structure manipulation, and more importantly, helps implement the undo/redo functionality on arbitrary operations. An undo operation requires inverting the corresponding operator, which can be complicated because of the consistency requirement. For example, the inverse operation of deleting multiple nodes would require recovery of all the neighbors of the nodes. Direct inference of such an inverse operation not only takes significant effort, but also leads to errors that can be difficult to track. After decomposing an operation into a sequence of elementary operations, we can construct the undo operation easily by reversing the sequence.

#### User interaction

The fundamental function of tracing software is changing neuron morphology with user inputs, which are usually composed of mouse clicks and key inputs. Since we defined an operation as the mapping of one set of nodes to another set, user interaction starts with node selection, which requires two components: SWC visualization and user input response. High-quality visualization of a neuron might be the most important feature of successful neuron editing. The ability to view the structures clearly greatly reduces examination time needed to identify errors. It is also necessary to provide both 2D and 3D views because each provides unique advantages. For example, a 3D view is well suited for displaying a global structure and a 2D view provides precise inference of dense local structures.

The most intuitive way to select a node is to move the mouse cursor to the node and then click. This requires mapping the screen cursor coordinates into the 3D SWC space. Multiple selections should also be supported to specify a set of nodes as the input of an operation. After selection, the user can trigger an operation with some input. So the operation becomes

$$f(S_{1}|\Theta )=S_{2}$$

where Θ is the set of parameters supplied from user input. For example, $f\left(\left\{n\right\}|\left(x,y,z\right)\right)=\{n,F_{p}(((x,y,z,r\left(n\right)),n_{0},n_{0},n_{0})|n)\}$ defines an operation of extending a branch from n to a node at $\left(x,y,z\right)$.

#### Create SWC nodes from image signal

For any standalone neuron-tracing software, it is essential to allow reconstructing neuron structure from raw image signals. In the SWC framework, this function can be formulated as

$$g(S_{1}|\Theta ,I)=S_{2}$$

where I is the image signal. Note that this actually defines a superfamily of SWC operations. The function is the same as an SWC operation if it is independent of I. An example of an image-dependent operation is shortest path creation, such as the one used by Simple Neurite Tracer (Longair et al., 2011), where $S_{1}=\{n_{i},n_{j}\}$ defines the source and target node and $S_{2}=\{n_{i},{n'}_{1},...,{n'}_{k},n_{j}\}$ forms the resampled shortest geodesic path from $n_{i}$ to $n_{j}$. The radii of ${n'}_{1},...,{n'}_{k}$, which are denoted as $r({n'}_{1}),...,r({n'}_{k})$ in the node definition, can be estimated automatically or linearly interpolated, depending on how the operation is defined.

### Software implementation

#### Architecture

**Figure 2:**
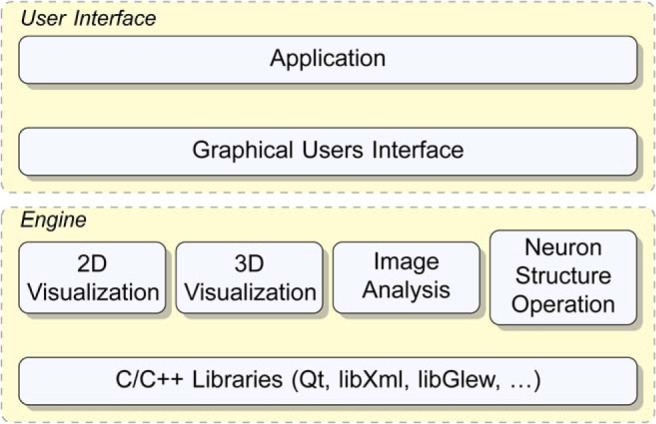
neuTube 1.0 is a GUI application built upon four major modules: 2D visualization, 3D visualization, image analysis, and neuron structure operation.

Based on the SWC framework, we have built neuTube 1.0 as a GUI application upon four core modules: 2D visualization, 3D visualization, image analysis, and neuron structure operation (Fig. 2).

##### 2D visualization.

**Figure 3:**
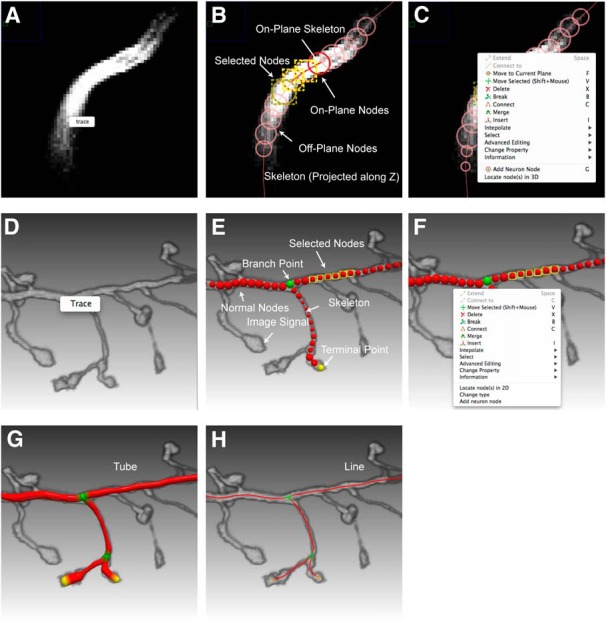
Tracing and editing interface of neuTube 1.0. ***A***, Interactive tracing in 2D view. ***B***, 2D view of SWC nodes. On-plane and off-plane nodes are distinguished by color saturation and transparency. A node with a yellow bounding box indicates that it is selected. ***C***, The context menu for editing in the 2D view, which can be triggered by a right mouse click. ***D***, Interactive tracing in 3D view. ***E***, 3D visualization of the tracing results. Branch nodes and terminal nodes are shown in green and yellow colors, respectively. Selected nodes are shown with their bounding boxes. ***F***, The context menu for editing in the 3D view. ***G***, A neuron shown as connected tubes. ***H***, A neuron shown as lines.

The 2D visualization module provides functions of displaying a 3D image and neuron structures slice by slice, as well as functions allowing the user to interact with the 2D display. This module facilitates close examination and precise editing. For example, driven by this module, the user can zoom into a region of interest to view details, locate tracing point precisely, or apply fine-tuning on a neuron structure. As the purpose of 2D visualization is to show the matching quality between the reconstruction and the data rather than a realistic neuron structure, we only used two geometrical primitives, lines and circles, to represent the morphology of a neuron (Fig. 3*B*). The 2D visualization is useful for showing the exact planar position of a node, yet not suitable for showing the position perpendicular to the plane. We used two strategies to address the issue. First, each node of the neuron is displayed as a circle when the plane cuts through the node. The circle is as large as the corresponding cross section of the node, informing the user by its size how far the node is from the plane. Second, we used colors to distinguish whether a node is centered on the current plane (on-plane) or not (off-plane): the node is shown with a fully saturated and opaque color when it is on-plane; otherwise the node color is semi-transparent and less saturated (Fig. 3*B*). The coloring options were tuned manually according to the user feedback and then used as immutable parameters of the software. To allow the user view the global structure of a neuron under reconstruction, we also project the whole skeleton onto the slice view, but with a thin and semi-transparent mode to minimize its interference with in-focus structures.

##### 3D visualization.

The 3D visualization module is designed to provide real-time rendering of 3D images and neuron structures. The user can perform tracing (Fig. 3*D*) and editing (Fig. 3*F*) in the 3D visualization window directly, in which any change in the neuron structure will be reflected in the 2D visualization window simultaneously, and vice versa.

This module supports both realistic neuron rendering and structural rendering by decomposing a neuron structure into three geometric primitives, including sphere, line, and conical frustum. The user can choose to view a neuron as connected spheres (Fig. 3*E*), tubes (Fig. 3*G*), or lines (Fig. 3*H*) for checking different morphological properties of the neuron. Besides the different view styles, the module also provides multiple color modes for inspecting topological properties of a neuron or dissecting multiple neurons.

#### Image analysis

This module offers automatic tracing of a neuron or a neuron branch to allow the user to obtain neuron structures with minimal interaction. For example, to select a branch, the user only needs to specify a point on the branch with one click. The algorithm and design were described in Zhao et al., (2011) and Kim et al., (2012). In this paper, one major improvement over the previously reported version (Kim et al., 2012) is the replacement of the cylindrical model by the tree model defined in the SWC framework. In addition, we have implemented a point-to-point tracing function based on the shortest path method used previously in automated reconstruction (Zhao et al., 2011). This function is similar to semi-automated tracing in the Simple Neurite Tracer and Vaa3d, but we have made it available in both 2D and 3D views by following the SWC framework.

#### Neuron structure manipulation

The module of neuron structure manipulation provides functions for the arbitrary editing of neuron nodes (Fig. 3*C*,*F*). The user can change the geometry and topology of a neuron structure with intuitive mouse clicks or keyboard shortcuts. This module supports operations described in the SWC framework and separates them into different levels.

We have also built high-level operations from elementary ones to reduce the labor required for structural operations. These operations are as follows.

##### Interpolate.

**Figure 4:**
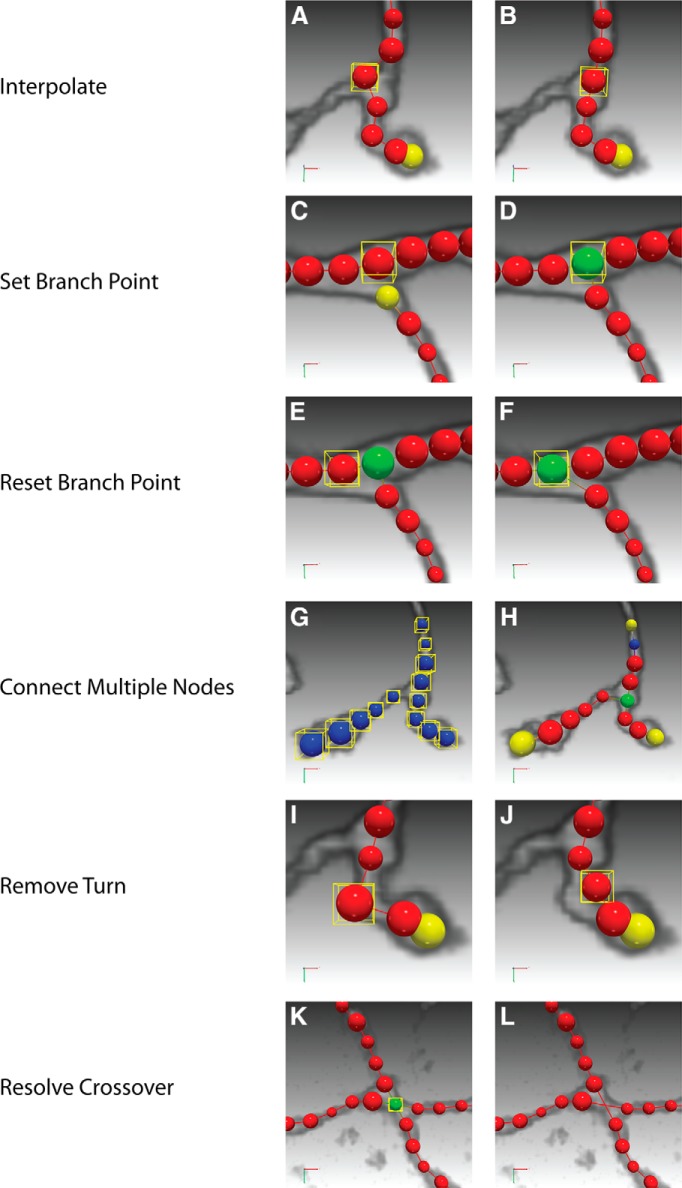
Examples of high-level operations of neuTube 1.0. To illustrate the operation, we visualize the nodes of a neuron in different colors according to the topology: blue for root nodes, green for branch nodes, yellow for leaf nodes, and red for other nodes. Selected nodes are highlighted by a yellow bounding box. For the corresponding operation as named in each row, the panels on the left (***A***, ***C***, ***E***, ***G***, ***I***, ***K***) show the selected nodes to operate and the panels on the right (***B***, ***D***, ***F***, ***H***, ***J***, ***L***) show the result of operation.

In many cases, a neuron branch or a segment thereof is smooth enough to be represented by piecewise linear structures. Interpolation takes advantage of this property and allows the user to quickly correct geometrical attributes of multiple nodes (Fig. 4*B*) by specifying the nodes that need interpolation (Fig. 4*A*).

##### Set branch point.

It often happens that a branch point is missed when the end of a segment is close to the interior of another segment. A completely manual editing operation would consist of selecting two nodes and joining them together. The operation of setting branch point simplifies this work by connecting the selected node (Fig. 4*C*) to the latest node in isolated branches when the connection creates a branch point (Fig. 4*D*).

##### Reset branch point.

This operation provides another way to correct a branch point. In this operation, the user selects a node (Fig. 4*E*) and the program will try to move the neighboring branching structure to the selected node (Fig. 4*F*). The program automatically determines which branch to move based on their angles.

##### Connect multiple nodes.

Connecting two nodes is one of the most basic operations, yet one that requires multiple steps, including selecting the nodes and triggering the connection command. When there are more and more nodes to connect, the number of human interactions increases proportionally. Therefore, neuTube 1.0 provides an operation for automatically connecting multiple nodes (Fig. 4*G*) by their edges in the minimal spanning tree of their pairwise distance graph (Fig. 4*H*).

##### Remove turn.

A turn is defined as three sequentially connected nodes that form an acute angle. The node in the middle is the turning point and the other two nodes are the flank nodes. The operation of removing a turn is to set the turning point (Fig. 4*I*) as the interpolation of the flank nodes (Fig. 4*J*). When the turning point is a branch point, the flank nodes are its two neighbors that form the sharpest turn.

##### Resolve crossover.

Crossover is a common tracing error in tracing when two branches are close at a certain point (Fig. 4*K*). Correcting a crossover requires several operations of connecting and breaking nodes. Therefore, we added an operation of automatic inference of crossover (Fig. 4*L*) to make the editing easier.

#### Implementation

The software is written in the C and C++ programming languages with several third-party libraries. The main third-party library is the Qt library (http://qt-project.org), which provides a cross-platform framework for GUI development. The 3D visualization module is built upon OpenGL 2.0 (http://www.opengl.org) and its shading language, GLSL (http://www.opengl.org/documentation/glsl). We developed a fast engine for rendering neuron structures by writing highly efficient shaders for two geometric primitives, sphere and conical frustum. The vertex shader finds bounding boxes of the geometric primitives on the screen, and then the fragment shader calculates ray-quadric intersections for each pixel inside the rasterized bounding box. All of our geometric primitives have adjustable opacity options and can be visualized in the order needed to generate a reasonable semi-transparent scene. For realistic rendering of complicated semi-transparent scenes, we have also implemented dual depth peeling and weighted average blending (Bavoil and Myers, 2008), which are two commonly used order-independent transparency methods. Since the two methods do not require special hardware features of high-end graphical cards, they provide neuTube 1.0 with the ability to render complicated scenes realistically without comprising the software portability. The user can switch from one method to the other in runtime to determine which one is better for the current scene.

To show an image signal in 3D, a volume, which contains the original image of the neurons to reconstruct, is uploaded to GPU as 3D texture and is rendered by a volume shader. The volume shader provides several volume composite methods, including direct volume rendering (DVR), maximum intensity projection (MIP) and its opaque variant, local maximum intensity projection (LMIP) (Sato et al., 1998). Each method has its own advantages. For example, MIP opaque allows the user to see weak signals that are typical of thin neural branches, LMIP is an extended version of MIP that can clearly depict spatial interrelations of neural branches, and DVR illustrates bright structures with low noise (Fishman et al., 2006). Users can also trace interactively in a 3D view by providing a seed point for tracing with a single mouse click, which represents a ray passing through the 3D volume. The seed point used for tracing is determined as the first location with maximum intensity along the ray.

## Results

**Figure 5:**
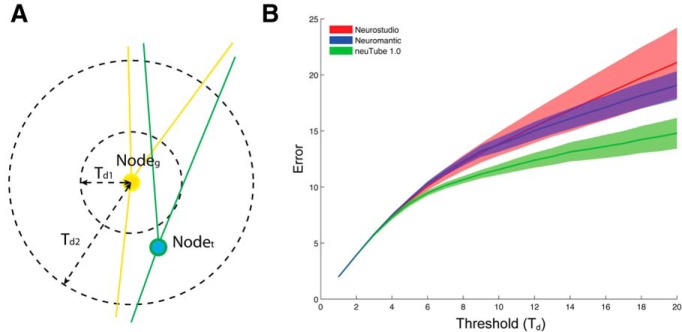
neuTube 1.0 helps produce significantly more accurate neuron structures than Neuromantic and Neurostudio do. ***A***, Node_g_ is a critical point from ground truth neuron and Node_t_ is a critical point from tracing result. These two points can be matched when $T_{d}=T_{d2}$ because the distance between them is less than $T_{d2}$. They cannot be matched when $T_{d}=T_{d1}$. ***B***, The solid curves show average errors measuring the discrepancy between the critical point sets from user reconstruction and ground truth under different distance thresholds. The surrounding envelopes are the 95% confidence intervals. The error curve of neuTube 1.0 (green) is consistently lower than the other two, with *p* < 0.01 (*t* test) when $T_{d}\geq 6$ (compared to Neurostudio) or $T_{d}\geq 5$ (compared to Neuromantic).

We compared our neuTube 1.0 to other neuron reconstruction software programs, namely, Neuromantic and Neurostudio. These two programs were chosen because their designs are close to the SWC framework, although they lack several important features available in the framework (Table 1). Four 3D images from the DIADEM datasets (Brown et al., 2011) were traced using all three software programs by four users given the same time constraint. Similar to the situation of real applications, the user could decide to stop tracing whenever he/she could not identify or fix an error. This reflects how well the software visualizes the reconstruction along with the data and the flexibility of the editing functions. The accuracy of tracing was measured by how well the critical points, including branching points and termini, were reconstructed compared to ground truth reconstructions. We extracted branching and terminal points as two point sets from each tracing result and matched them to the ground truth by solving the linear assignment problem (LAP) using the Jonker-Volgenant Algorithm (Jonker and Volgenant, 1987). Assuming there are a total of N points with M of them matched to the ground truth, the reconstruction error is calculated as:

$$Error=\frac{T_{d}\left(F_{p}+F_{n}\right)+\sum_{m=1}^{M}d_{m}}{N}$$

where $F_{p}$ and $F_{n}$ are the number of false positives and the number of false negatives, respectively, *T_d_* is the maximal distance allowed between two matched points (Fig. 5*A*), and $d_{m}$ is distance between the mth matched pair of points. In this calculation, the term $T_{d}\left(F_{p}+F_{n}\right)$ is the cost of missing critical points and $\sum_{m=1}^{M}d_{m}$ is the cost of position offset.

**Table 1:** Feature comparison of neuTube 1.0 with Neuromantic and Neurostudio

Software	Undo/Redo	2D editing	3D editing	3D image interaction	2D neuron visualization	3D visualization
neuTube 1.0	Unlimited	Yes	Yes	Yes	Slice-by-slice	Volume and neuron structure
Neuromantic	1 step	Yes	Limited^*b*^	No	Slice-by-slice	Neuron structure
Neurostudio	1 step	Limited^*a*^	Limited^*b*^	No	Projection	Volume and neuron structure

*^a^* Cannot change node size.

*^b^* No topological operation.

Our error metric is designed on the basis of the DIADEM metric (Gillette et al., 2011), but with two major modifications for better evaluation of interactive neuron reconstruction. One modification is that our metric matches critical points globally, while the DIADEM metric matches critical points in a certain order, which starts from the root position and may give an upstream node more importance. For user editing, missing an upstream node and missing a downstream one usually mean the same type of error. Our matching method is order-independent and treats these nodes equally. The other different feature of our metric is the combination of topological errors and position errors, with the introduction of the matching threshold (*T_d_*), as the weight of mismatches. The threshold *T_d_* is similar to the threshold region of the DIADEM metric, but we do not assign it a fixed value, which is often subjective or application dependent. Instead, we define the error metric as a function of *T_d_*.

By comparing scores across a wide range of threshold values, we showed that neuTube 1.0 achieved consistently better reconstruction accuracy than Neuromantic and Neurostudio (Fig. 5*B*). The advantage of neuTube 1.0 is more significant when the threshold is larger, indicating that neuTube 1.0 helps the user obtain more accurate neuron structures by identifying more critical points than the other two software tools.

### Application example

**Figure 6:**
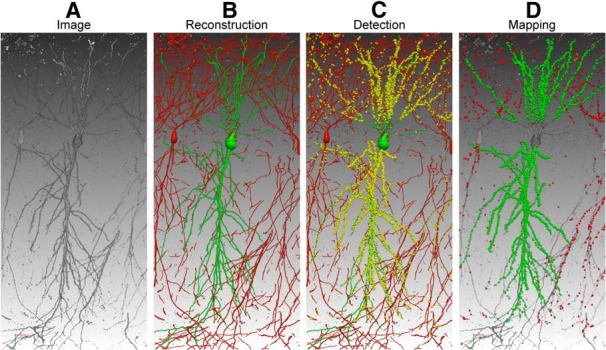
Mapping brain connectivity with neuTube 1.0. ***A***, The original 3D confocal image contains postsynaptic neurons. ***B***, The target neuron (green) was traced semi-automatically. The red branches belong to background neurons. ***C***, mGRASP-labeled synapses (yellow) were detected automatically, with sizes enlarged for better visualization. ***D***, The synapses were mapped to the target neuron (green) and background neurons (red).

We have used neuTube 1.0 to map the fine-scale synaptic connectivity between hippocampal regions (CA3–CA1) of the mouse brain (Druckmann et al., 2014). To analyze the spatial synaptic connectivity pattern, mammalian GFP reconstitution across synaptic partners (mGRASP) (Kim et al., 2012) was used to label the synapses, and red fluorescence protein (i.e., dTomato) was used to label the postsynaptic dendrites (Fig. 6*A*). neuTube 1.0 was used to reconstruct 3D structures of postsynaptic neurons (Fig. 6*B*). In our application, we detected the mGRASP-labeled synapses using our mGRASP detection package (Feng et al., 2012) (Fig. 6*C*), and then assigned each synapse to a reconstructed neuron by calculating its intensity-weighted distances to all nearby neurons (Fig. 6*D*). To make the mapping more accurate in the step of synapse assignment, we need to reconstruct not only the selected neurons but also the remaining dendrite branches or background neurons (Fig. 6*B*) because the distance to the nearest selected neuronal branch alone can mis-assign synapse puncta (Feng et al., 2014). A practical solution to this is to reconstruct all dendrite branches from the 3D image first and then edit the target neurons, which must be reconstructed correctly. neuTube turned out to be the right tool for this problem because the SWC framework specifies that the software can start the reconstruction from any SWC file. With the help of neuTube 1.0, we have built a fine-scale mapping of the hippocampal CA3–CA1 circuit and, with further statistical analysis, revealed spatially structure and clustered synaptic connectivity patterns between CA3 and CA1 (Druckmann et al., 2014).

## Discussion

We designed the SWC framework and implemented it in neuTube 1.0 (www.neutracing.com) to improve the efficiency of reconstructing neuron structures accurately. Guided by the framework, the software combines 2D/3D visualization, semi-automated tracing algorithms, and flexible editing options to simplify the task of neuron reconstruction. The SWC framework is not designed to solve the problem of high-throughput neuron tracing, which is different and more challenging. As revealed by the recent DIADEM competition (Liu, 2011), a reliable and generally applicable high-throughput neuron-tracing tool may not be available in the near future. While waiting for the ideal solution, neuroscientists will benefit from better neuron reconstruction tools. Therefore, the goal of the SWC framework is to provide a general architecture, which can adopt state-of-the-art image analysis methods and modern software techniques, for building better interactive neuron reconstruction tools.

Our framework has one limitation, which is that it can only produce neuron structures defined in the SWC format. However, this is usually not a significant concern because the SWC model suffices for most purposes, such as comparing neuron shapes, performing Sholl analysis, uploading neuron structures to NeuroMorpho.org (Ascoli et al., 2007), and simulating neuron activities. Many researchers prefer the SWC format rather than more complicated models because it helps to avoid overfitting to imaging artifacts: the resolution of optical microscopy is usually not high enough to reveal fine details. Even when a structure more complex than the SWC model is needed, reconstructing the neurons in the SWC model is still useful as an initial input for later shape refinement (Evers et al., 2005).

Our experiment showed that the results from neuTube 1.0 were generally better than those from Neurostudio (Myatt et al., 2012) and Neuromantic (Wearne et al., 2005), but it is still worth noting the strengths of these software programs. Neuromanic allows multi-tile tracing to reconstruct neurons from more than one field of view. This is particularly useful for reconstructing a large neuron that requires horizontal stage movement to cover all branches. Neurostudio offers only limited free editing functions, but its ability to trace multiple branches from one seed point is a very useful feature to reduce labor, and its intrinsic radius estimation based on rayburst sampling (Rodriguez et al., 2006) can be implemented in any other software to refine the neuron structure.

As the functions of multi-branch tracing and rayburst radius estimation naturally fit in the SWC framework, we plan to include them in a future upgrade of neuTube 1.0. Additionally, there are ongoing efforts to extend the software to broader applications, including tracing neurons in bright-field images and analyzing neuron morphologies, such as identifying neuron types from electron microscope reconstructions (Zhao and Plaza, 2014).

Because a user can import results from other software into neuTube 1.0 to do further editing, neuTube 1.0 is also a complementary tool to other automated or interactive neuron tracing tools. For instance, the Vaa3d software has added neuTube 1.0 as a plug-in in recent releases (vaa3d.org). Also, other developers can improve their own software by adopting the SWC framework. To facilitate any such adoption, we have made the source code of neuTube 1.0 available at https://github.com/janelia-flyem/NeuTu.

## Synthesis

The decision was a result of the Reviewing Editor Alfonso Araque and the peer reviewers coming together and discussing their recommendations until a consensus was reached. A fact-based synthesis statement explaining their decision and outlining what is needed to prepare a revision is listed below. The following reviewers agreed to reveal their identity: Pedro Larrañaga and Maurizio De Pitta

Present manuscript presents a new software tool called "neuTube 1.0" for realistic reconstruction of neuron morphology. The authors present a complete first version of the software and it is expected that the software will be further developed in the future to grow in capabilities. In addition it offers a friendly and intuitive interface for the great benefit of potential future users.

### Reviewer # 1

The manuscript describes neuTube 1.0, an open-source software for efficiently reconstruct neurons from flourescence image data. I have some issues on the current version of the paper:

Page 5, line 12: x<sub>i, y<sub>i, z<sub>i and r<sub>i for x,y,z,r. Please explain the meaning of x<sub>i, y<sub>i, z<sub>i and r<sub>i.

Page 5, line 15: K<sub>1 and K<sub>n should be k<sub>1 and k<sub>n.

Please refer to the software as neuTube 1.0, avoiding the term "neuTube".

Page 6, line 3: explain why redundancy in the representation is a good idea.

The symbol <b>n' in the three equations of lines 14-17 at page 6, should be explained.

page 8, line 18: .... is the vector containing the parameters??

Page 10, Figure 3: please organize the subfigures A to H according to Section 2.2.2 and Section 2.2.3 respectively.

Page 12, line 10: .... were described in Zhao et al.(2011) and Kim et al. (2012).

Page 13, line 24: Therefore, neuTube 1.0 provides an operation for automatically.

Page 15, line 14: Please comment on LMIP.

Page 16, equation between lines 8 and 9: Error = ...... This allows to an easier reading of Figure 4.

Page 17, Figure 4: Please provide neccessary details to understand the meaning of the Figure. For example, how the errors of the 4 human users on the 4 3D images from DIADEM have been computed.

Page 17, Figure 4: To scientifically prove the superior behavior of neuTube 1.0 over Neurostudio and Neuromantic, it shoul be shown that the differences are statistically significants. That is, please provide the result of the corresponding hypothesis test.

In the reference by Sato et al. (1998), please write the name of the journal in capital letters.

### Reviewer # 2

The paper by Fen and collaborators, present a new software tool "neuTube 1.0" aimed at efficiently reconstructing neuron morphology from light-microscopy images. The tool, although at its early stage of development, is based on SWC data - likely the standard format for neuromorphometric data - and has the premises of good portability. In addition it offers a friendly and intuitive interface for the great benefit of potential future users. The paper is generally well written and suitable for publication. I have essentially only minor corrections to suggest to the authors, which I am following detailing:

#### Abstract

L6: You shall mention what the SWC format is, for readers that are not familiar with this software. At least mention that this is a standard data format in neuromorphometric data. Provide accordingly proper reference (like the neuromorpho archive).

L11: Mention explicitly the other two software tools.

#### Significance Statement

L15: The [novel] software we present...

L17: replace "synchronized" by "in parallel".

#### Introduction

L3: mapping [of] brain circuits.

L7-L9: this sentence is somehow cumbersome please rephrase. Also consider merging it with the sentence in L5-L6.

L10: the concept of "intuitive" operation is ambiguous.

L13: With these criteria [in mind], we [may] identify

L15: [which] focuses

L18-L22: this sentence is too long and lacking of proper bibliographic reference.

#### Materials and Methods

Figure 1. Expand caption providing an essential description of the workflow.

L13 (and elsewhere): use quotation marks or italics to first define "parent", "children" or "sibling".

P5,L15: replace "ancestor" by parent.

P5,L20-L22: I would define S1 and S2 before the formula.

P6,L4-L18: there is some confusion on the notation here. You originally define operations on sets, but then you rewrite f_p,f_c,f_s as operations on single nodes defined as vectors (or tuples). Moreover, you switched from symbol T_i for nodes to "n". I would use only one symbol to improve clarity.

P6,L5: the concept of "sibling" was not previously introduced.

P6,L8,L9: use quotation marks or italics for "geometrical/structural operations".

P6,L12-L19. The overall meaning of this sentence and the presentation of the operations is hard to grasp. What do you mean by "multiple" levels to construct operations? How do you set these levels?

P8,L18: \theta is the [vector of] parameters

P9,L2: this function can [be] formulated as

P9,L9: resampled

P9,L10: what do you mean by radius of a node. I might have missed it, but I could not find any definition in the text. I assume that the radius is that of the equivalent sphere or circle used to trace each branch, but then you shall explain it.

P11,L13: We use color saturation etc. Please cite relevant bibliography here if the process is automatic. Otherwise specify that saturation and color transparaency are by manual adjustment.

P12,L12: ...replacement of the cylindrical model [by]...

P12,L15: cite relevant sources for the shortest path algorithm.

P12,L22: "...framework, and separate them into different levels."

P13-P14: when talking about all the high-level operations, it would be nice to have a figure (akin of Figure 3) that illustrate each of these operations.

P13,L1-L3: Replace this sentences by: "We have also built high-level operations... These operations are following listed."

P13,L5: In many cases, a neuron [branch or a segment thereof] is...

P13,L14: in isolated branches [when] the connection...

P13,L16: This operation provides another way...

P13,L21: replace 'but' by 'yet'.

P14,L18: cite relevance reference for OpenGL and GLSL.

P15,L2-L5: "For realistic rendering of complicated scenes, we also implemented two order-independent transparency methods like the ... and the...". Also describe briefly the reason of your choice and the advantages of implementing these methods.

P15,L14: cite relevant bibliography.

#### Results

P16,L9: where F_p and F_n are ... respectively, T_d...

P16,Table 1 caption: "Feature comparison of neuTube 1.0 with Neuromantic and Neurostudio.

P17, Figure4 and related text: It is not clear what the "Threshold" is. Perhaps adding a graphical example could help otherwise make sure to better clarify in the text.

P17,L6-L8. Why did you choose specifically this error measure? Was this these same measure used in the other softwares? It's true that users may obtain neuron structures by identifying more critical points... However, identifying more critical points might now be easy and result in a time-consuming task for users. How do you argue this aspect too against the other software tools.

P18, Figure 5: use yellow spheres or another contrasting color instead of cyan spheres for mGRASP/dTomato synaptic puncta. The current color choice makes almost impossible to distinguish synapses from neuronal spheres.

P18,L17-L19: This sentence is not fully clear. How mapping of postsyanaptic branches comes at play in the consideration of biological constraints such as spine length?

P19,L7: Drop version number from neuTube.

#### Discussion

P19,L9: I would avoid mentioning "preliminary version" as it is a confusing technical detail.

P20,L17,L18,L23: cite relevant reference for companion softwares.

Finally, I would also like to see in the discussion mention of any future plan to extend neuTube capabilities (if any) such as for example the inclusion of features that companion softwares have (like Neurostudio's ability to trace multiple branches, automatic branch recognition etc.).
